# A swine model of soy protein–induced food allergenicity: implications in human and swine nutrition

**DOI:** 10.1093/af/vfz025

**Published:** 2019-06-25

**Authors:** John Scott Radcliffe, Luiz F Brito, Lavanya Reddivari, Monica Schmidt, Eliot M Herman, Allan P Schinckel

**Affiliations:** 1 Department of Animal Sciences, Purdue University, West Lafayette, IN; 2 Department of Food Science, Purdue University, West Lafayette, IN; 3 The School of Plant Sciences, The University of Arizona, Tucson, AZ

**Keywords:** allergy, soybeans, swine

**Implications**

- Basic digestive processes result in the breakdown of most foodborne antigens; however, a small proportion of food-derived antigens cross the intestinal barrier leading to a brief period of hypersensitivity that is usually followed by the development of oral tolerance.
- A shift from oral tolerance to sensitization marks the potential for clinical allergy development.
- The anatomical, physiological, histological, genomic homology, and immunological similarity between pigs and humans make pigs a better model than traditional rodent species to study food allergies and intervention strategies.
- A subset of pigs naturally develop soy allergies making them an ideal model for soy allergies.

## Introduction

**Figure 1.**
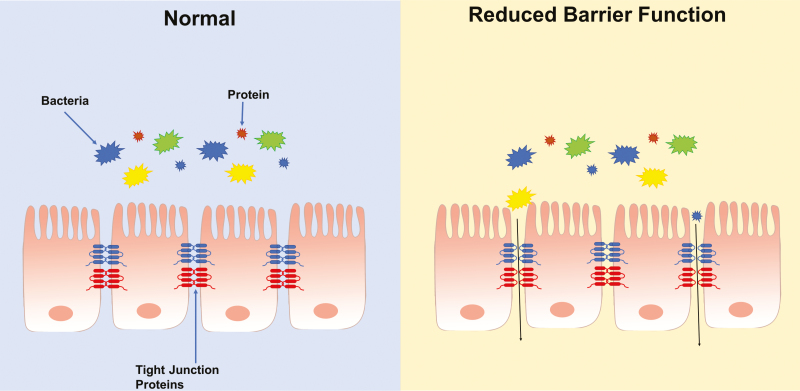
Intestinal barrier function is maintained by a series of tight junction proteins, which connect adjacent cells and prevent bacteria and protein from traversing the paracellular space. Reductions in gut barrier function potentially allow bacteria and proteins to travel between cells. It has been hypothesized that reductions in gut barrier function may result in increased incidences of food allergy development. Conversely, food allergy development has also been reported to reduce gut barrier function.

Food allergies are of concern in human nutrition and are a growing concern or focus in companion animal nutrition, but have received little attention in production animal agriculture. For companion animals especially some breeds of dogs, hyper-allergenic food has been commercialized to provide an avoidance diet. For production animals, feed-induced hypersensitivity has been documented and has economic impact on swine and calves. The timing of introduction of potentially allergenic feed components is a critical consideration in swine production as to not impair the animal’s growth rate and economic performance. Severity of responses to food allergens can range from mild to anaphylaxis and death. Responses can increase, decrease, or disappear over time. The threshold antigen quantity needed to elicit a response varies by allergen and individual, but as little as 1 mg is sufficient to induce a fatal response to peanut in some people. In general, there is a dose response for allergens but with increasing exposures the dose required for a strong response decreases. In humans (Sicherer and Sampson, 2014) and other animals, the highest prevalence of food allergies is observed in the neonate and is correlated to the relative immaturity of the gastrointestinal tract and disturbances in gut barrier function (Figure 1) and may be further amplified by gut stress. Gut barrier function is a broad topic in and of itself because impaired gut barrier function has a role in development of food allergies (Groschwitz and Hogan, 2009), and the food allergic response may further reduce gut barrier function (Chen et al., 2014), potentially having long-term effects on the onset of other enteric diseases and overall gut health. There is extensive literature on the development and management of food allergy, but some basic questions such as why some proteins are allergenic while closely related proteins from other species are not allergenic, remain to be answered. This paper is not a detailed review of food allergies, but rather an overview of current knowledge regarding soy food allergies, with a focus on the pig as a model for the study of soy allergies and with additional information on how soy allergies and use of soybean-sourced feed can affect production animal agriculture.

### Incidence of food allergies

Globally, incidence and recorded incidence of food allergies are on the rise. The increase in food allergies is most notable in urban populations with increasing prosperity but is independent of the population’s geographic origin and therefore its genetic background. Infants are considered the most at risk population and highest allergen rates are reported in industrialized nations (Loh and Tang, 2018). However, accurate incidence rates are difficult to acquire because the reporting population is also those who are more likely to have available medical attention. Other factors at play include the globalization of new and novel foods some of which introduce new food allergens into a previously naïve population, such as the experience of introducing Kiwi fruit to the United Kingdom population beginning in the 1970s. The clinical standard for food allergy confirmation is a food challenge. However, this assessment is resource intensive and places patients at risk that would ideally take place in a setting where prompt intervention is available if anaphylaxis results. The incidence of reporting food allergies often relies on self-reporting, which tends to over-estimate incident rates and for many individuals to mistake other types of responses for true IgE-mediated food allergy. In most self-assessments rates 5-fold or higher claim food allergy than is confirmed by clinical assessment. In a review of global food allergies, Loh and Tang (2018) cited food allergy incident rates, based on food challenges, in children ranging from 3.6% to 10% for western countries and from 1% to 7.7% in Asia. In the United States, an estimated 4–6% of children manifest food allergies. There is an estimated additional 2 million people suffering from ulcerative colitis, Crohn’s disease, celiac disease, food protein–induced endocolitis syndrome, or other chronic enteric disorders in the United States.

**Figure 2.**
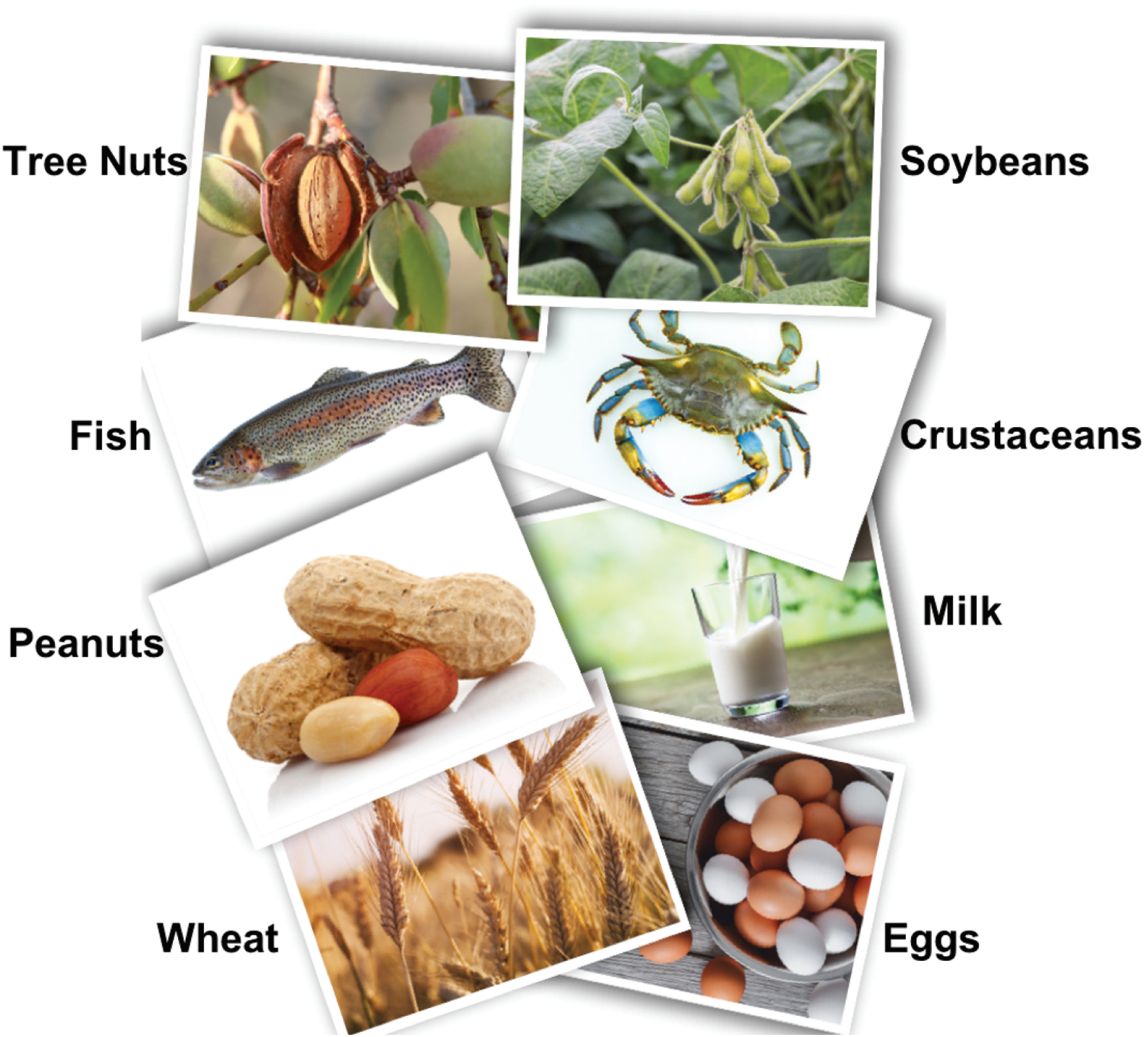
The 2004 Food and Allergen Labeling Consumer Protection Act (FALCPA) regulated the inclusion of eight primary foods responsible for 90% of all documented food allergies by requiring clear identifiable labeling to assist consumers to avoid problematic foods. These eight allergens are milk, eggs, fish, shellfish (Crustaceans), tree nuts, peanuts, wheat, and soybeans.

The 2004 Food and Allergen Labeling Consumer Protection Act (**FALCPA**) regulated the inclusion of eight primary foods responsible for 90% of all documented food allergies by requiring clear identifiable labeling to assist consumers to avoid problematic foods. These eight allergens are milk, eggs, fish, shellfish (Crustaceans), tree nuts, peanuts, wheat, and soybeans (Figure 2). Soybeans are particularly problematic because of their widespread inclusion in processed foodstuffs which greatly limits consumer choices even if properly labeled. An additional primary use of soybeans is in infant formulas as a replacement for milk protein, and therefore, with the exception of milk, soybeans are the most likely FALCPA-listed allergen to be ingested by infants. With possible wide-spread exposure at an early age, it is surprising that soybean food allergenicity receives less attention than many of the others on the FALCPA list. This lack of emphasis likely results from the fact that initial allergic responses tend to be less severe and more antigen (soy protein) is required to elicit a response (Sicherer and Sampson, 2018). Soybean exposure most often induces atopic skin reactions and gastrointestinal distress but rarely produces the fatal anaphylaxis associated with peanut and tree nut exposure. Sensitized individuals often become tolerant to soybeans over time, often as toddlers or young children while peanut allergy often persists throughout life (Sicherer and Sampson, 2018). From the perspective of swine production, soy feed hypersensitivity is of economic significance as soy protein is a nearly ubiquitous component of swine diets in many areas of the world. The inclusion of soy in pig diets is often delayed until several weeks after birth and postweaning because of its adverse impact on growth. Although the soy allergic response can be mild and generally decreases with age, a growing body of evidence suggests alterations in gut-barrier function early in life predisposes an individual to a variety of diseases later in life (Medland et al., 2016). However, it is something that could be overlooked in a production setting. Therefore, there is a critical need to study food allergies in swine production, while at the same time, swine are a clinically relevant animal model for food allergy research in humans.

### How does a food allergy develop?

Food allergies occur when an ingested protein (that is otherwise benign to consume) crosses the intestinal barrier and sensitizes the immune system so that when it is again ingested, a hypersensitive response results. The definition of an antigen is “any substance (such as an immunogen or a hapten) foreign to the body that evokes an immune response either alone or after forming a complex with a larger molecule (such as a protein) that is capable of binding with a product (such as an antibody or T cell) of the immune response” (Meriam-Webster, 2019).

Normally, potential antigens in feed are broken down by digestive enzymes in the stomach and small intestine. However, injury, disease pressure, or gut immaturity may result in the uptake of antigens that are often resistant to digestion via transcellular or paracellular absorption routes (Price et al., 2013). Additional evidence suggests that the immune system functions to routinely sample potential luminal antigens at a rate of about 2% that likely plays a key role in the development of food intolerance and allergy. This process of antigen sampling involves dendritic cells and may involve antigen presentation by goblet cells and M cells (Johnston et al., 2014).

Once absorbed, the body can either become tolerant of foodborne antigens or it can become sensitized. Oral tolerance occurs after antigen presentation to regulatory T cells by macrophages, dendritic cells, or other antigen presenting cells, and is mediated through IL-10 producing T cells and IgA producing B cells. Poorly defined perturbations in this tolerance pathway result in sensitization, characterized by a dominant type 2 T-helper cell-mediated response (Th2) resulting in increased antigen-specific IgE production by B cells. Following sensitization, re-exposure to the antigen results in an antigen-specific IgE-induced degranulation of mast cells with subsequent histamine release. In reality, the process of oral tolerance or sensitization is extremely complicated and involves a myriad of molecules that may influence or help switch the process from tolerance to sensitization or vice versa. A detailed overview of the collective knowledge of sensitization is presented by Sampson et al. (2018).

## Soy Allergenicity

The first documented case of soy allergenicity in humans was reported in 1934 by Duke (1934). Since then, more than 28 different soy antigens have been identified, which bind to IgEs from people with soy allergy. Of these, three soy proteins appear to be responsible for the majority of reported soy allergies, including Gly m Bd 30k, Gly m Bd 28k, and Gly m Bd 60k. Gly m Bd 30k (also referred to as P34) was discovered as a highly immunogenic protein in soybean (Kalinski et al., 1990, 1992) and was correlated with dominant IgE binding. In individuals with atopic soybean allergy, P34/Gly m Bd 30k was the dominant soy allergen with positive IgE binding in greater than 65% of individuals tested with an atopic soy allergy (Ogawa et al., 1991). In other assessments of human infant soybean allergy Helm et al. (1998) showed that P34/Gly m Bd 30k is the immunodominant dominant allergen. P34/Gly m Bd 30k is a minor soybean seed protein comprising less than 1% of the total protein (Kalinski et al., 1992) and is a member of the Papain superfamily of cysteine proteases that also includes dominant allergens of pineapple, kiwi fruit, papaya, and dust mites. A unique characteristic of P34/Gly m Bd 30k is that the active site cysteine is mutated to glycine and it appears to be a nonfunctional protease that has been conserved and is present in all domesticated and wild-ancestral relatives of soybean (Yaklich et al., 1999). The function of P34/Gly m Bd 30k remains unknown. A biotech silenced null mutation has been produced and there was no impact of the absence of the allergens on seed ontogeny or seed composition. A frame-shift null mutation has been identified in the USDA’s National Soybean Collection and that null as well as its introgression into a model agronomic line similarly showed no impact on the seed. A biotech over-expression of P34/Gly m Bd 30k that increased accumulation of the protein from less than 1% total to 8–10% of total protein again showed no apparent impact on the seed’s ontogeny. That P34/Gly m Bd 30k null mutations have no agronomic impact presents the opportunity to use this material to produce soybeans with increased biosafety. Aside from the potential human use to eliminate P34/Gly m Bd 30k from soybeans used for infant formula and food, P34 has also been shown to cross the intestinal epithelium of piglets creating the circumstance for induced hyper-sensitivity.

Soybean has other allergens that often produce positive atopic responses albeit with less intensity than P34/Gly m Bd 30k. Among these allergens, Gly m Bd 28k is also referred to as P28 in the literature and is a seed glycoprotein belonging to the Cupin protein superfamily. Cupin proteins have a distinct core consisting of a beta-barrel domain, which is believed to be highly stable, and may result in increased allergenicity (Candreva et al., 2016). Positive IgE binding for Gly m Bb 28K has been observed in 25% of individuals tested with an atopic soy allergy (Ogawa et al., 1991). Gly m Bd 60k is the alpha subunit of beta-conglycinin, which is a major storage protein in soybeans. Positive IgE binding for Gly m Bb 60K has been observed in 25% of individuals tested with an atopic soy allergy (Ogawa et al., 1991).

One curious aspect of soybean protein in comparison to the other seven FALPCA proteins is that the soybean threshold can require 100-fold more protein to elicit a response in comparison to any of the other FALCPA proteins (Cordle, 2004). However, the amount needed to elicit a response is still relatively small at ~400 mg (safe dosage for 90% of allergic patients, from Cordle, 2004) and would be a minor part of any meal for an at-risk infant or immature animal. It should also be noted that there is a wide variation in the amount of protein capable of inducing an allergic response. There is no dose that should be considered safe in sensitized individuals. Although infrequent, there are documented incidences of soybean-induced anaphylaxis.

## Models of Soy Hypersensitivity

Two dominant models have been established for studying food allergies: 1) the oral sensitization model and 2) the hyperimmunization model (Cordle, 2004). In the oral sensitization model, animals are generally fed diets containing the protein of interest (soy) for approximately 1 mo followed by a systemic challenge using the same protein. If animals were sensitized during the oral challenge, then an IgE-mediated response will occur with varying intensity from mild atopic to life-threatening anaphylaxis reactions. In the hyperimmunization model, animals are injected with the protein of interest, solubilized in an adjuvant, usually in a fairly aggressive immunization protocol and then the appearance of antibody titers in the blood is measured.

Controlled experiments of food allergies in humans are problematic needing rigorous control of diet, and when sensitization occurs, includes the risk of anaphylaxis and death. Animal models are critical for food allergy research (Van Gramberg et al., 2013). Mice have been used extensively to study food allergy due to the robust resources associated with their physiology, biology, and genetics (including the availability of numerous relevant inbred lines and the capacity to produce gene knockdowns). As the best characterized animal system, mice have diverse molecular and genomic tools available to the public. However, mice come from highly inbred lines and therefore do accurately represent the range of outcomes across a more genetically diverse population of humans. Mice do not easily develop food allergy and mice have significant differences in gut physiology relative to humans; thus, mice are a relatively poor proxy of the at-risk human infant. Mice tend to develop oral tolerance fairly quickly, which has caused researchers to focus on strains of mice that are more prone to display a type 2 T-helper cell response, and/or to use an adjuvant that stimulates the type 2 T-helper cell response.

A better proxy for humans would be a large omnivore model with a gut physiology that closely mimics the human anatomy. Pigs are intriguing as a food allergy model for humans because the anatomy, physiology, and histology of pigs are more similar to humans than rodents are to humans. Food allergies are also naturally present in a subset of the swine population, and these allergies tend to decrease with age, which is similar to the human clinical experience. The pig has been used previously as a model for egg (Rupa et al., 2008) and peanut allergies (Stanley et al., 2002). In both cases, pigs were sensitized with intraperitoneal injections of egg or peanut proteins given in conjunction with cholera toxin, which drives the Th2 response. Both studies reported significant wheal and flare reactions following a skin prick test in sensitized animals. Antigen-specific IgG was detected in sensitized animals and evidence in both studies was indicative of an IgE-mediated allergenic response. However, the lack of commercially available specific antiserum to swine IgE limits confirmation of these results.

**Figure 3.**
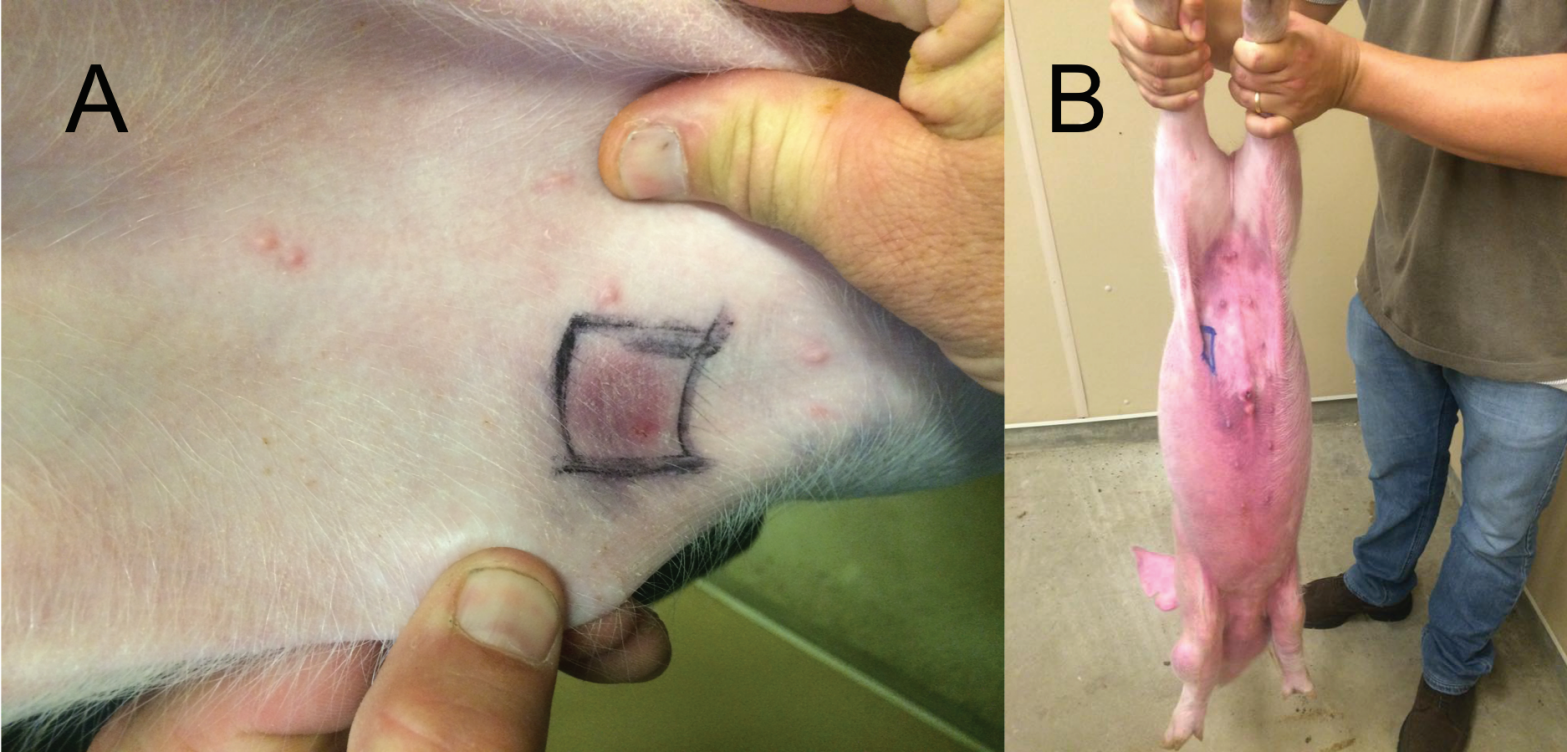
(A) Classic wheal (swelling) and flare (redness) response following a skin allergy test in sensitized pigs selected for hypersensitivity to soy protein. (B) Whole body atopic rash in a soy hypersensitive pig following a soy skin allergy test.

Soy hypersensitivity has potential implications in commercial swine production because soybean meal as a protein nutritional source is ubiquitous across pig diets in the United States and much of the world. In 1989, Heppell et al. (1989) described an IgG-mediated immune response in newly weaned pigs fed soybean meal, which led to oral tolerance. In 1990, Li et al. (Li et al., 1990) described a transient hypersensitivity to soy protein resulting in elevated soy-specific IgG, reduced intestinal villi height, and reduced growth performance. In 2012, Calbrix et al. (2012) described two divergently selected pig populations for hyper- and hypo-sensitivity to soy protein. Unlike, previous swine models of food allergies, this model utilized selective breeding for more than eight generations to divergently select for populations of pigs that were either hyper- or hypo-sensitive to soy protein challenge. In each generation, pigs were orally sensitized to soy protein in the diet following weaning and then subjected to a skin prick test using soy protein. The significance of the selection protocol is that this closely mimics exposure resulting from food/feed-producing sensitization and then assayed much as a food intolerant human patient would be by atopic response. Wheal and flare scores were used to characterize the severity of the allergic response and in the selection of matings to produce each subsequent generation. Pigs selected for more than eight generations for sensitivity to soy protein have systemic atopic and physiological responses (skin test, gut morphology, and leakiness) similar to that seen in the soy allergy response observed in human neonates (Figure 3). Growth rate and feed intake are impaired in the hypersensitive line in the 2 wk postweaning (Ferreira et al., 2014). In addition, *GLP2, Occludin, and p65/RelA* gene expression are reduced in the hypersensitive line, indicating perturbations in intestinal growth, gut barrier function, and immune response (Amaral et al., 2018). Histologic examination further demonstrates a disruption in gut-barrier function as evidenced by extensive damage to the intestinal epithelia of soy hypersensitive pigs with blunted and/or sheered villi. Proteomic analysis of the jejunum reveals that proteins involved in cytoskeletal structure, cell-cell junctions and adhesion, the exosome, and vesicular secretions all exhibit enhanced abundance in the line of pigs selected for hypersensitivity to soy protein compared with pigs selected for hyposensitivity to soy protein. Similar to humans, tolerance seems to increase with age, even in the hypersensitive line. Recently, using the predicted amino acid sequence of swine IgE (based on the published nucleotide sequence; Vernersson et al., 1997), Calbrix et al. (2012) developed monoclonal antibodies to two unique sites and used these antibodies in a sandwich ELISA assay. Data using this assay confirm a strong IgE-mediated response in swine genetically selected for hypersensitivity to soy protein, following sensitization and then an oral challenge. Hashimoto-Hill et al. (2019) hypersensitized both the high- and low-soy sensitivity lines using a repeated intraperitoneal injection of soy protein and cholera toxin and then orally challenged both lines with soybean meal in the diet. They observed reduced villi heights and greater mucosal degradation, indicative of a higher inflammatory response in pigs selected for hypersensitivity to soy protein over eight generations. Challenging sensitized pigs from the hypersensitive line with soy protein resulted in an apparent movement of Th2 cells from circulation to mesenteric lymph nodes and the jejunum. This increase in gut-associated Th2 cells was accompanied by an increase in GATA3, which is a transcription factor produced by Th2 cells and innate type 2 lymphoid cells.

Food allergy ontogeny is complex and the mechanistic details are well beyond the scope of this article. Beyond base exposure and sensitization, several factors including individual genetics, environment, microbiome, and diet all control an animal’s immune response from one of oral tolerance to one of sensitization. Soy protein food allergies in swine and humans tend to be less severe than tree nuts and peanuts that share its FALCPA designation. Soybean hypersensitization reactions are usually confined to gastrointestinal disturbances and skin rashes with more severe symptoms (e.g., respiratory distress or cardiac arrhythmias) being relatively uncommon. Most pigs appear to go through a short period of soy hypersensitivity followed by development of oral tolerance. Soy-feed induced hypersensitivity often is overlooked or dismissed as an economic issue in production animal agriculture. There is a growing body of evidence suggesting that early life stressors in humans and animals increase the susceptibility of other gastrointestinal disorders or predisposition for disease later in life (Medland et al., 2016). A variety of animal models have been used to study early life stress including, but not limited to, neonatal maternal separation, limited nesting stress, neonatal colonic inflammation/distension, early weaning stress, and transport stress. One of the consistent results of all of these models is a disruption of gut barrier function. This is significant because the reduction in gut barrier function can increase the rate of antigen transfer across the gut barrier through disorganized intestinal tissue increasing the incidence of food allergies. One of the many etiologies of food allergies is a reduction in gut barrier function, and food allergy might serve as an early life stressor affecting subsequent health issues during the life cycle.

### Swine model for comparative genomic analyses

Another area of research under current investigation refers to the use of the swine model to understand the genetic mechanisms and underlying biology of the food allergy response by identifying genomic markers, genes, and metabolic pathways associated with the soy allergy response. Genetics has a crucial role in the likelihood of developing food allergy hypersensitivity (Cochrane et al., 2009; Hong et al., 2009). However, little is known about the genomic regions associated with food allergies and disorders of the gastrointestinal tract. As previously discussed, dietary compounds can influence the animals’ genome in order to alter the expression of genes, transcription factors, gene products (e.g., proteins and metabolites), and the gut microbiome (e.g., Frese et al., 2015). The recent advent of high-throughput “omics” technologies has created an opportunity to integrate multiple levels of biological information (genome, transcriptome, metabolome, and microbiome) to better understand the individual response to dietary compounds (e.g., soybean-produced EGF and L-glutamine) and the potential interactions between genomic markers and the diet (e.g., nutrigenomics). The effect of selected dietary products on inflammatory response, oxidative stress, and metabolism in humans, based on large-scale profiling of genes, proteins, and metabolites, has been shown to be a useful approach to better understand disease outcomes (Bakker et al., 2010). Furthermore, the gut microbiome can also be partially regulated by the host genome.

Classical rodent models have been useful for understanding the basic biology of genes and proteins involved in human diseases, but the usefulness of rodents is restricted to specific diseases (Walters et al., 2017). It has been shown that the conservation of synteny between the human and pig genomes is three times greater than between humans and mice (Johansson et al., 1995; Sun et al., 1999; Bakker et al., 2010). Analysis of porcine genomic sequences has indicated an almost identical gene content to human sequences, but with some gene-order differences (Sun et al., 1999). Furthermore, selection pressure in pigs is more similar to humans (Groenen et al., 2012) and protein structure in pigs and humans is more similar compared with mice (Dawson et al., 2017). This highlights a greater likelihood of identifying important genes associated with soy allergy in swine that will have similar effects in humans. For instance, Dawson et al. (2017) reported that swine are a scientifically acceptable intermediate species between rodents and humans to model immune function relevant to humans. The authors concluded that there were more similar rates and classes of genes in humans and pigs than in mice and they supported the use of swine to model human immunological and inflammatory responses.

In our research group, two lines of swine were divergently selected for soybean allergy response for more than eight generations. Soy allergy response was measured by challenging pigs with a high soybean meal content diet (28%) for 21-d postweaning, followed by intradermal skin testing. Reactions were used to select young pigs to establish high- and low-reacting lines of pigs (Calbrix et al., 2012). The large number of pigs that can be phenotyped (as soybean is a common diet ingredient) and that are currently genotyped using whole-genome genotyping platforms (for breeding purposes) will increase the power of identifying genomic regions and metabolic pathways associated with the food allergy response and intestinal disorders. Therefore, pigs represent a great animal model to investigate food allergy in humans. Meurens et al. (2012), discussing about the use of the swine model for infectious diseases, stated that “over the next few years there is no doubt that the pig model will be increasingly accepted as the alternative large animal model to the well-established mouse model.”

## About the Authors

**John “Scott” Radcliffe** is an Associate Professor in the Department of Animal Sciences at Purdue University. He received his BS, MS, and PhD from Virginia Tech and was briefly on the faculty in the Department of Animal and Poultry Sciences at Virginia Tech prior to moving to Purdue University. Dr. Radcliffe has two fundamental research interests: 1) environmental nutrition—how do we feed pigs to ensure the highest level of environmental sustainability? and 2) gut health. Within those two areas, Dr Radcliffe’s work can be partitioned into four categories: 1) reduction of the environmental impact of production animal agriculture (primarily swine) through increasing the efficiency of nutrient utilization and reducing nutrient excretion; 2) use of animal models to study nutrient absorption/utilization in human disease states; 3) gut health and function; and 4) the immune response in food allergy development and food-related digestive disorders.

**Luiz F. Brito** is an Assistant Professor of Quantitative Genetics and Genomics in the Department of Animal Sciences at Purdue University. He also holds an Adjunct Faculty position in the Department of Animal Biosciences at the University of Guelph (Canada). Luiz earned a BSc degree (with distinction) in Animal Sciences and a MSc in Genetics and Animal Breeding at the Federal University of Viçosa (Brazil). He completed his PhD and a postdoctoral fellowship at the Centre for Genetic Improvement of Livestock, University of Guelph (Canada). Luiz is broadly interested in quantitative genetics, genomics, and systems biology applied to genetic improvement of livestock species. His research group focuses on genetics and genomics of complex traits associated with animal behavior and welfare, environmental efficiency, and adaptation to challenging environments.

**Lavanya Reddivari** is an Assistant Professor in the Department of Food Science at Purdue University. She earned her doctoral degree from Texas A&M University with an emphasis on genetic and environmental regulation of phytochemicals. As a USDA postdoctoral fellow, she focused on understanding the processing effects on phytochemical composition and function using the pig as a model system. Dr Reddivari’s research now focuses on dietary phytochemicals and the interaction of gut bacteria in health and disease. Her lab is specifically interested in understanding the anti-inflammatory efficacy of plant bioactive compounds; how plant bioactive compounds influence the gut bacterial composition and function; the role of gut bacterial metabolism of dietary phytochemicals in modulating colonic inflammation and maintaining gut barrier integrity.

**Monica Schmidt** is an Associate Professor in the School of Plant Sciences and the BIO5 Institute at the University of Arizona. Her PhD in Genetics is from the University of British Columbia. She was a post-doctorate associate at both the University of Georgia and the USDA at the Danforth Plant Science Center. Her research interests are in both functional foods and functional genomics. Her research applies molecular biology and genetic techniques to help alleviate major agricultural problems, primarily in seed traits in soybean and maize.

**Eliot M. Herman** is a Professor of Plant Sciences in the Bio5 Institute at the University of Arizona. He has a BA and MA from UCSB and a PhD from UCSD. He has held prior positions at USDA/ARS and at the Donald Danforth Plant Science Center. His research interests include improving soybean protein content and quality including food allergens to optimize soybean as a source of food and feed. He was awarded the 2004 Plow award from the U.S. Secretary of Agriculture for his work on eliminating soybean allergens.

**Allan P. Schinckel** is a Professor of Quantitative Genetics in the Department of Animal Sciences at Purdue University. Dr. Schinckel earned a BSc degree (with distinction) in Animal Sciences at Iowa State University, followed by a MSc and PhD in Genetics and Animal Breeding at the University of Nebraska-Lincoln. Dr. Schinckel is broadly interested in quantitative genetics, animal compositional growth modeling, and the modeling of environmental effects (health status, immune system activation, and stressors) on animal growth. His research focuses on the genetic improvement of swine and the means in which genetic selection affects the animal’s nutritional and environmental requirements. Dr Schinckel is responsible for selection of the high and low food allergic lines of pigs.
